# In Vitro Anticancer and Antibacterial Activities of the Essential Oil of Forsskal’s Basil Growing in Extreme Environmental Conditions

**DOI:** 10.3390/life13030651

**Published:** 2023-02-26

**Authors:** Ammar Bader, Ashraf N. Abdalla, Najla A. Obaid, Lamees Youssef, Hind M. Naffadi, Mohamed E. Elzubier, Riyad A. Almaimani, Guido Flamini, Ylenia Pieracci, Mahmoud Zaki El-Readi

**Affiliations:** 1 Department of Pharmacognosy, Faculty of Pharmacy, Umm Al-Qura University, Makkah 21955, Saudi Arabia; 2 Department of Pharmacology and Toxicology, Faculty of Pharmacy, Umm Al-Qura University, Makkah 21955, Saudi Arabia; 3 Department of Pharmacology and Toxicology, Medicinal and Aromatic Plants Research Institute, National Center for Research, Khartoum 2404, Sudan; 4 Department of Pharmaceutics, Faculty of Pharmacy, Umm Al-Qura University, Makkah 21955, Saudi Arabia; 5 Medical Genetic Department, Faculty of Medicine, Umm Al-Qura University, Makkah 21955, Saudi Arabia; 6 Department of Biochemistry, Faculty of Medicine, Umm Al-Qura University, Makkah 21955, Saudi Arabia; 7 Dipartimento di Farmacia, University of Pisa, Via Bonanno 33, 56126 Pisa, Italy; 8 Department of Biochemistry, Faculty of Pharmacy, Al-Azhar University, Assiut 71524, Egypt

**Keywords:** *Ocimum forskolei*, *Ocimum forsskaolii*, essential oil, GC-MS, HCT116 cancer cell line, apoptosis, cell cycle, bacteria, antimicrobial agent

## Abstract

Many species belonging to the genus *Ocimum* are used for aromatic, medicinal, and cosmetic purposes. The essential oil (OFEO) obtained by hydrodistillation of the flowering aerial parts of Forsskal’s Basil “*Ocimum forskolei* Benth” growing in extreme environmental conditions in Mecca Region, Saudi Arabia was analyzed by GC-MS. The main constituents were phenylpropanoids (methyl eugenol 55.65% and eugenol 11.66%), monoterpene (linalool 9.75%), and sesquiterpenes (germacrene D 3.72% and β-caryophyllene 2.57%). The OFEO was tested against MCF7, HT29, and HCT116 cancer cells and compared with normal fibroblast cells (MRC5). The MTT assay showed that HCT116 was more sensitive to OFEO (IC50 5.34 μg/mL), which reduced the number of HCT116 colonies at 6 μg/mL, while causing complete colony death at 12 and 24 μg/mL. Western Blotting and qRT-PCR were used to evaluate the level change of different proteins with respect to GAPDH. OFEO upregulated the apoptotic protein (caspase 3), and downregulated the cell proliferation proteins (AKT and pAKT), cell cycle arrest (PCNA, Cyclin D1), and the anti-apoptotic Bcl2 proteins. OFEO was also tested against reference strains of Gram-negative and Gram-positive bacteria including *Escherichia coli*, *Klebsiella pneumonia*, *Pseudomonas aeruginosa*, and *Staphylococcus aureus* by using the well-diffusion and assessing their MICs, which ranged from 250 to 500 μg/mL.

## 1. Introduction

Plants belonging to the genus *Ocimum* are of great economic and medicinal value, as many species are used for their nutritional, pharmacological, and detoxicant properties [1,2,3]. *Ocimum basilicum* L., “Sweet basil”, the most nutritious species of this genus, and its cultivars are distributed worldwide and are largely used in the food and perfume industries [4,5]. Several studies were performed on *Ocimum* species essential oils, extracts, and pure constituents, revealing potent anti-proliferative and antimicrobial activities [6,7,8]. Besides the essential oil, some non-volatile secondary metabolites isolated from *Ocimum* species have shown anti-cancer activity [9,10]. Caffeic acid, isolated from *O. gratissimum* L., had an anti-proliferative effect against human cervical cancer cells (HeLa), and it dramatically increased the apoptosis of HeLa cells through the activation of various caspases [9]. Moreover, ursolic acid isolated from an *O. basilicum* extract, induced a significant decrease of cell percentage in the anaphase/telophase stages of the cell division cycle. Ursolic acid also showed effects on F-actin and microtubules [10]. Regarding the essential oil of *Ocimum* sp., it possesses a marked antimicrobial activity against a broad range of bacteria and fungi because of the presence of diverse classes of volatile constituents, such as monoterpenes, phenylpropanoids, and sesquiterpenes [11,12].

Cancer is a multifactorial disorder characterized by uncontrolled cell proliferation that outwits the body’s defenses and traditional anticancer treatments. It is globally one of the main causes of death: in 2020, 19.3 million new diagnosed cancer cases and 9.9 million cancer deaths were recorded [13]. Breast, lung, colorectal, prostate, and stomach cancers are the most frequent types [13]. New medicines and therapeutic systems have been developed for cancer treatment. The inhibition of cell death, the increase of DNA damage, the overexpression of P-glycoprotein, and the epithelial–mesenchymal transition are among the mechanisms by which tumor cells might gain innate or acquired multidrug resistance [14]. In the search for new anticancer drugs, plants are considered a rich source of possible metabolites to be used in anticancer therapy; it is no coincidence that many of the best anticancer substances are of natural origin [15].

Besides cancer, infectious diseases cause high mortality worldwide, mainly in developing countries, requiring the discovery of new sources of antimicrobial agents from medicinal plants [16,17,18,19,20,21,22,23]. Essential oils are a complex mixture of volatile secondary metabolites with diverse bioactivities, including both anticancer and antimicrobial ones [24].

In the present study, we selected the essential oil of *Ocimum forskolei* Benth, also known as *Ocimum forsskaolii* (Lamiaceae), to investigate its biological properties. This is an aromatic plant widely distributed in Saudi Arabia. It is a bushy-woody herb, with bright green leaves and lilac-pink flowers, which grows at altitudes from 300 to 1400 m. It is used by the native people in Saudi Arabia to aromatize butter and tea, as well as for its carminative and insect-repelling properties. Moreover, its flowers are a good source of propolis [25]. Previous studies of OFEO revealed the chemical diversity of volatile constituents depending on the geographical origin; the essential oil obtained from Omani sample of *O. forskolei* flower contains mainly estragole (59.4–65.2%), followed by linalool (25.0–28.1%) [26]. The Kenyan sample was rich in fenchone 49.86%, Limonene 14.08%, camphor 5.93% and α-Caryophyllene 4.46%, respectively [27], while the Ethiopian sample was rich in linalool 17.3%, methyl chavicol 19.3% and (E)-methyl cinnamate 33.0%, or myrcene 24.2% and eugenol 25.0%, respectively [28]. In previous study on *O. forsskaolii*, the silver nanoparticles of the aqueous extract of the leaves revealed an antimicrobial effect [7]. The most predominant classes of compounds in the genus *Ocimum* are phenylpropanoids, monoterpenes and sesquiterpenes [29,30,31,32,33]. The aim of the present study was to investigate the essential oil composition of the flowering aerial parts of *O. forskolei* growing wildly in Saudi Arabia and to assess its anticancer effect through cytotoxicity and molecular target validation using reverse transcription polymerase chain reaction (qRT-PCR) and Western Blot (WB). In addition, the essential oil of *O. forskolei* was tested against Gram-positive and Gram-negative microorganisms responsible for urinary tract infections, such as *Staphylococcus aureus*, *Klebsiella pneumonia*, *Escherichia coli*, and *Pseudomonas aeruginosa*.

## 2. Materials and Methods

### 2.1. Materials

All the chemicals were purchased from Sigma Aldrich, Riyadh, Saudi Arabia, unless otherwise mentioned.

### 2.2. Methods

#### 2.2.1. Plant Material

The flowering aerial parts of *O. forskolei* were collected at Wadi Rahjan, near Makkah Al-Mukarramah in February 2021 (Google Earth GPS coordinates: 21°17′52″ N 40°05′23″ E); the plant was identified by one of the authors A. B., and a voucher specimen, number SA-IT-2021/1, was deposited at the Herbarium of Pharmacognosy Lab, Faculty of Pharmacy, Umm Al-Qura University.

#### 2.2.2. Essential Oil Hydro-Distillation

Fresh plant material (200 g), previously cut into small pieces, was hydro-distilled with a Clevenger standard apparatus for 2 h (yield: 0.52%). Aliquots of the obtained essential oil were diluted to 10% in HPLC-grade n-hexane prior to the GC-MS injection, while the remaining parts were stored in a freezer at −18 °C until biological testing.

#### 2.2.3. Gas Chromatography-Mass Spectrometry Analyses and Peak identification

Gas chromatography–electron impact mass spectrometry (GC-EIMS) analyses were performed with an Agilent 7890B gas chromatograph (Agilent Technologies Inc., Santa Clara, CA, USA) equipped with an Agilent HP-5MS (Agilent Technologies Inc., Santa Clara, CA, USA) capillary column (30 m × 0.25 mm; coating thickness 0.25 μm) and an Agilent 5977B single-quadrupole mass detector (Agilent Technologies Inc., Santa Clara, CA, USA). The working conditions of essential oil analysis were similar to our previous work [18]. The temperature of the oven was set to rise from 60 °C to 240 °C at 3 °C/min. Temperatures were set as follows: injector temperature, 220 °C; transfer-line temperature, 240 °C. The carrier gas was He with 1 mL/min flow. The acquisition was performed with the following parameters: full scan, with a scan range of 35–300 *m*/*z*; scan time: 1.0 s; threshold: 1 count. The identification of the constituents was based on the comparison of their retention times (tR) with those of pure reference samples and of their linear retention indices (LRIs) relative to the *n*-alkanes (C9–C25) series. The detected mass spectra were compared with those listed in the commercial libraries NIST 14 and ADAMS, as well as in a homemade mass-spectral library built up from pure substances and components of essential oils of known compositions and MS literature data [34,35,36,37,38].

#### 2.2.4. Cell Culture

Three cancer cell lines, MCF7 (human breast adenocarcinoma), HT29 (human colorectal adenocarcinoma), and HCT116 (human colorectal carcinoma), were used, together with MRC5 (normal human fetal lung fibroblast). All cells were obtained from the ATCC. The three cancer cell lines were sub-cultured in RPMI-1640 media (10% FBS); while MRC5 cells were maintained in Eagle’s minimum essential medium (EMEM, 10% FBS), all at 37 °C, 5% CO_2_, and 100% relative humidity.

#### 2.2.5. Cytotoxicity and Selectivity Studies

The cytotoxicity of OFEO was evaluated by MTT assay, as previously reported [39]. The cell lines were separately cultured in 96-well plates (3 × 10^3^/well), and incubated at 37 °C overnight. Final extract or doxorubicin concentrations were 0–100 μg/mL (DMSO 0.1%; *v/v*, *n* = 3). Plates were incubated for 72 h and then MTT was added to each well. The absorbance was read with a multi-plate reader (BIORAD, PR 4100, Hercules, CA, USA). Optical density of the purple formazan A550 was proportional to the number of viable cells. Extract concentration causing 50% inhibition (IC_50_) compared with control cell growth (100%) was determined using GraphPad Prism (San Diego, CA, USA). The selectivity index (SI) for the extract was calculated by dividing its IC_50_ against MRC5 cells/IC_50_ against the other tested cells.

#### 2.2.6. Clonogenic Survival Assay

The clonogenic survival assay, which studies the ability of individual cancer cells to form colonies, was performed according to a previous report [40]. HCT116 cells were seeded in 2 mL of low-density medium (2 × 10^2^) in 6-well plates (*n* = 2). Plates were incubated at 37 °C overnight to allow attachment. Cells were treated with OFEO (0, 6, 12, and 24 μg/mL). After 72 h, the medium was discarded, and 2 mL of fresh medium was added. Plates were checked under the microscope every 2 days. After 14 days, clusters of at least 50 cells were considered a colony. The medium was aspirated and the cells were washed with cold phosphate-buffered saline (PBS), and then fixed with cold methanol for 5 min at room temperature. Cells were then stained with 0.5% *v/v* methylene blue in methanol: H_2_O (1:1) for 15 min. Colonies were washed with PBS and H_2_O. Next, plates were left to dry before the final counting of colonies.

#### 2.2.7. Western Blotting

In this study, the level change of AKT, pAKT, PCNA, cyclin D1, Bcl2, and Caspase 3 proteins was investigated using Western Blotting. The effect of OFEO (0, 6, 12, and 24 μg/mL) on HCT116 cells was tested following a previous reported method [41]. Briefly, HCT116 cells (1 × 10^6^ cells/well in a 6-well plate) were treated for 24 h. The HCT116 cells were lysed with a mixture of RIPA lysis and extraction buffer in ice for 10 min. The lysates were centrifuged at 14,000× *g* for 10 min at 4 °C. BCA assay was used to quantify protein concentration. Samples with 20 μg of total proteins were separated on 10% SDS-PAGE gels and transferred to PVDF membranes. Membranes were blocked for 1 h in TBS-T containing 5% non-fat dry milk and incubated with antibodies at 4 °C overnight. After washing, blots were incubated with secondary antibodies (HRP-conjugated) for 1 h. Target proteins were detected using the LI-COR system for enhanced chemiluminescence. The following antibodies were used: AKT, pAKT, PCNA, cyclin D1, Bcl2, Caspase 3, and GAPDH in 1:1000 dilutions. Secondary anti-rabbit HPR conjugated antibodies (1:3000 dilution) were used.

#### 2.2.8. Quantitative Real-Time PCR (qRT-PCR)

The HCT116 cells were treated with 12 μg/mL of OFEO for 24 h. Total RNA was extracted using a Purlink RNA isolation kit. Briefly, RNA (1 μg) was subjected to qRT-PCR using cDNA kits and a qPCR master mix kit according to the manufacturer’s instructions. Quantitative PCR (qPCR) was performed to evaluate the expression of eight genes, including total AKT, phosphorylated AKT (pAKT), PCNA, cyclin D1, Bcl2, and Caspase 3, while GAPDH was utilized as a housekeeping gene. The qPCR reactions were carried out in triplicate on an Applied Biosystems™ 7500 FAST Real-Time PCR Systems using SYBR green qPCR master mix with 100 ng cDNA template and 0.4 M of target-specific primers (Table 1). The following conditions were employed in the qPCR reaction: initial denaturation at 95 °C for 90 s, followed by denaturation at 95 °C for 20 s, annealing at 51 °C for 15 s, and extension at 60 °C for 30 s for 45 cycles. The expression of the genes was normalized against GAPDH, and the CT value was used to compute the relative gene expression level [42].

#### 2.2.9. Antibacterial Assay

The antibacterial activity of OFEO was evaluated against the following reference bacteria: *Escherichia coli* (ATCC 25922), *Klebsiella pneumoniae* (ATCC13883), *Pseudomonas aeruginosa* (ATCC 27853), and *Staphylococcus aureus* (ATCC 29213). All strains were stored in tubes containing glycerol (20%) at −20 °C, then sub-cultured on nutrient agar (Saudi Prepared Media Laboratory Company Ltd., SPML Ltd., Dammam, Saudi Arabia).

The well diffusion method was performed using Muller–Hinton agar (Saudi Prepared Media Laboratory Company Ltd., SPML Ltd. Dammam), which was previously described as a well-established method for investigating the antimicrobial activity of plant extracts [43]. Briefly, the bacterial inoculum (0.5 McFarland) for each strain was spread uniformly on Muller–Hinton agar (SPML Ltd. Dammam). The prepared solution of OFEO was added (100 μL) into wells (6 mm diameter) by cutting holes/wells in the agar gel. Each well was made 20 mm apart from the others. Antibiotic discs (Amikacin and Amoxicillin, Bioanalyse Tibbi Malz. San. ve Tic. Ltd., Ankara, Turkey) were used as positive controls. The plates were then incubated for 24 h at 37 °C. After incubation, bacterial growth inhibitions were determined by measuring the zone of inhibition (in millimeters) surrounding the treated wells and compared with that of the positive control. The experiment was repeated in triplicate.

The minimum inhibitory concentration (MIC) assay was performed by using a microdilution method on a 96-microliter plate [44]. Briefly, microdilutions were performed for each bacterial isolate used in this study, which was prepared in nutrient broth and standardized to be diluted to 0.5 MacFarland (equal to 1 × 10^8^). The first well contained 100 μL of OFEO (starting inoculum 1000 mg/mL) + 100 μL nutrient broth, which were prepared to start the dilution. The MIC value was obtained employing a microdilution method by adding the starting concentration (1000 μg/mL) from each prepared solution which was then diluted for the subsequent wells (500, 250 and 125 μg/mL) by mixing, and then 100 μL was transferred from each starting well into the subsequent well, which initially contained 100 μL nutrient broth. Dilution continued for each well (three subsequent wells). Then, 100 μL of bacterial suspension (1 × 10^8^) that was prepared for each bacterial isolate was added to each well (in triplicate) including the organism control (inoculum with nutrient broth only) to compare the growth turbidity; negative control wells were included in triplicate and contained the nutrient broth with 1000 μg/mL OFEO. Positive control wells were prepared using 1000 μg/mL of ciprofloxacin solution (≥98% HPLC, Sigma-Aldrich). The 96-well plates were then incubated aerobically at 37 °C for 24 h, and the antimicrobial effect of the solutions was clearly evidenced by a clear/turbidity effect.

#### 2.2.10. Statistical Analysis

Graphpad Prism was used to perform multiple comparison tests ANOVA (one-way) with Tukey’s post-hoc for the assessment of statistical differences.

## 3. Results

### 3.1. OFEO Analysis

To the best of our knowledge, the chemical composition of the *O. forskolei* essential oil (OFEO) from samples growing wildly in Saudi Arabia was here reported for the first time, and analysis results are reported in Table 2. Overall, 33 compounds were detected, covering 100% of the whole composition. Phenylpropanoids, representing 68.7%, were undoubtedly the major detected chemical class, with methyl eugenol (55.6%) and eugenol (12.1%) as the most abundant constituents. Sesquiterpenes were also found in relevant percentages, even though their hydrocarbon form prevailed (14.2%). Among this class, the most representative components were germacrene D and β-caryophyllene, both detected in amounts below 5%. Among sesquiterpenes, also the sulfide derivative mint sulfide was identified, and it was detected in the genus *Ocimum* for the first time.

**Figure 1:**
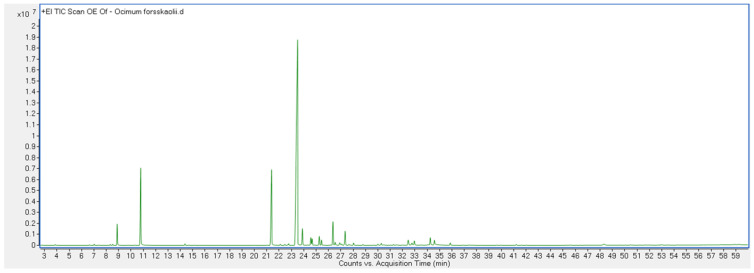
Chromatogram of GC analysis of OFEO.

Finally, another important class was that of oxygenated monoterpenes, covering 10.0% of the whole composition. This class, however, was almost totally represented by linalool (9.8%), which was the third highest constituent found in the analyzed EO. The chromatogram of GC analysis is shown in Figure 1.

### 3.2. Cytotoxicity and Selectivity Studies

The cytotoxicity of OFEO was evaluated against MCF7, HT29, and HCT116 cell lines and exhibited variable activities (IC50: 5.34–17.09) (Table 3). The colon cancer cells (HT29 and HCT116) were three-fold more sensitive to OFEO compared with the breast cancer cells (MCF7). The IC50 of OFEO was comparable to that of doxorubicin against HCT116 cells. The extract showed selectivity against the three cancer cells compared with MRC5, while it was more selective against HCT116 cells (Table 4).

### 3.3. Clonogenicity Assay

**Figure 2:**
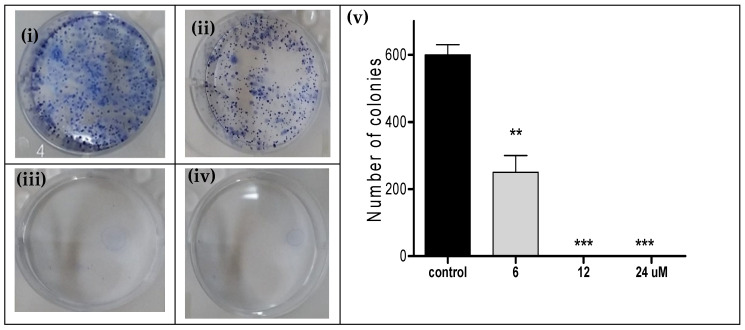
Clonogenicity of HCT116 cells following 72 h treatment in a six-well plate with (**i**) vehicle control, (**ii**) OFEO (6 μg/mL), (**iii**) OFEO (12 μg/mL), and (**iv**) OFEO (24 μg/mL). (**v**): Bar chart showing the four treatments (*x*-axis) against the number of HCT116 colonies (*y*-axis). Experiments were repeated three times. Statistical differences compared with untreated control cells were assessed by one-way ANOVA with the Tukey’s post-hoc multiple comparison test (*p* < 0.010 (**), *p* < 0.001 (***) were taken as significant).

The clonogenicity of OFEO extract was tested against the most sensitive cell line, i.e., HCT116 cells. The concentrations tested were IC_50_, 2 × IC_50_ and 4 × IC_50_ (i.e., 6, 12, and 24 μg/mL). The extract decreased the number of HCT116 colonies at 6 μg/mL, while it caused complete colony death at 12 and 24 μg/mL Figure 2.

### 3.4. Western Blotting

**Figure 3:**
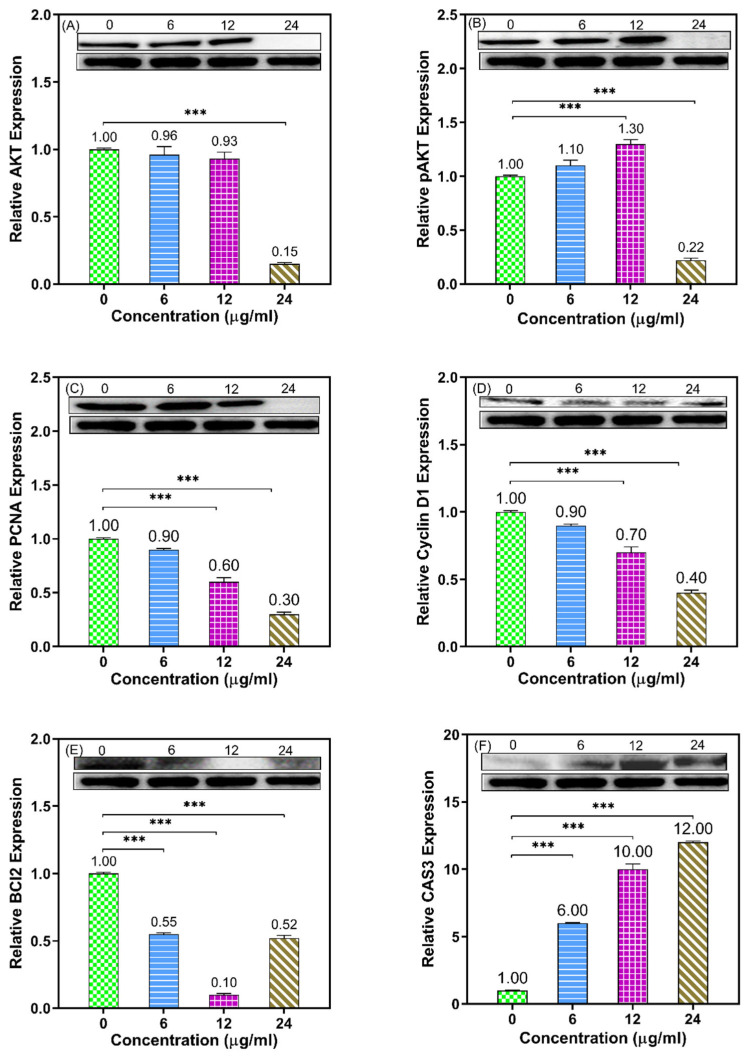
Western blotting analysis showing the effect of OFEO (0, 6, 12, and 24 μg/mL; 24 h) in cultures of HCT116 regarding eight proteins: AKT (**A**), pAKT (**B**), PCNA (**C**), CyclinD1 (**D**), Bcl2 (**E**), and Caspase 3 (**F**) compared with GAPDH as the reference protein (bottom blots). Bar graphs represent the quantitative densitometric value of the expressed protein vs. GAPDH and refer to mean values calculated from three experiments (*** *p* < 0.001 vs. control).

Following the identification of HCT116 cell line as the most sensitive to the OFEO, the possible underlying cell proliferation/survival, cell cycle, and apoptosis molecular mechanisms of cell death were investigated. Immunoblotting can be used to identify the up- and down-regulated proteins based on different concentrations of tested essential oil. Figure 3 represents the relative gene expression of studied genes related to GAPDH as reference protein. Caspase 3 was up-regulated, while the AKT, pAKT, PCNA, Bcl2, and cyclinD1 were down-regulated.

### 3.5. Quantitative Real-Time PCR (qRT-PCR)

**Figure 4:**
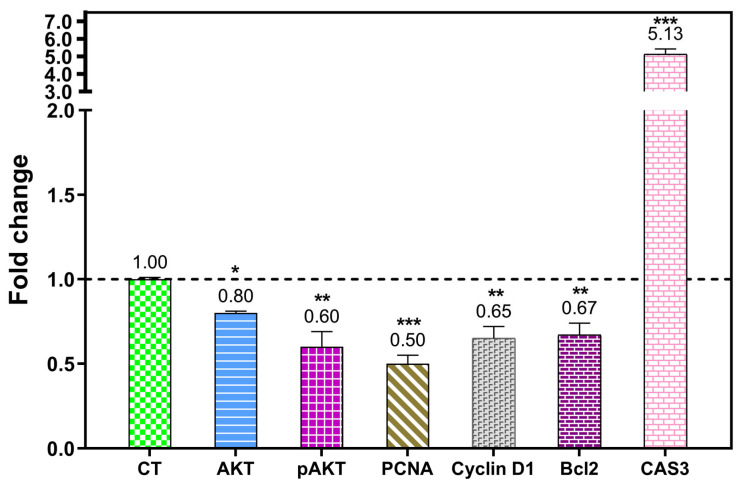
qRT-PCR results showing fold change in the gene expression of (decrease): AKT, phosphorylated AKT, PCNA, cyclin D1, and Bcl2. Caspase 3 increased in HCT116 cells treated with OFEO (12 ug/mL, 24 h). Gene expression was related to GAPDH as a housekeeping gene, and was normalized to the untreated cells (control, CT = 1-fold change as shown by the dashed line). *p* < 0.100 (*), *p* < 0.010 (**), and *p* < 0.001 (***) were taken as significant.

To confirm whether pre-treatment with OFEO influences the gene expression of cell cycle and apoptosis pathways in HCT116 cells, qRT-PCR was performed. As shown in Figure 4, the results revealed that OFEO at 12 ug/mL (24 h) upregulated the expression levels of Caspase 3, while it downregulated pAKT, PCNA, Bcl2, and Cyclin D1, all compared with GAPDH. These results indicated that treatment of HCT116 cells with OFEO inhibited the cell proliferation, caused cell cycle arrest, and induced apoptosis in HCT116 cells.

### 3.6. Antibacterial Activity

#### 3.6.1. Well-Diffusion Method

The zones of inhibitions were recorded and illustrated in Table 5. The diameter of inhibition of the growth occurred from the diffusion of the solution applied into the agar media. We compared the zone of inhibition of OFEO solution with the positive control diameter. The OFEO showed medium inhibition for *E. coli*, *K. pneumonia*, *P. aeruginosa*, and *S. aureus* (15, 15, 15, and 13 mm, respectively) and with Amikacin (18, 20, and 22 mm).

#### 3.6.2. Minimum Inhibitory Concentration (MIC) Assay

The MIC is the lowest concentration of the solutions prepared of OFEO that showed inhibition of the growth of the tested bacterial strains and appeared as a clear solution. Microdilution of the solution concentration of OFEO started from 1000 μg/mL and then was subsequently diluted to make 500, 250, and 125 μg/mL. OFEO inhibited the growth of all bacterial strains at 250 μg/mL except *P. aeruginosa*, which was inhibited at 500 μg/mL as shown in Table 6.

## 4. Discussion

The investigated plant grows in the Makkah region in extreme environmental conditions, characterized by a long, sweltering, and arid summer, with maximum temperatures around 45 °C in June and minimum temperatures around 29 °C in the same month; the winter is short, comfortable, dry, and mostly clear. By means of GC-MS, three main classes of compounds (phenylpropanoids, monoterpenes, and sesquiterpenes) were identified in the OFEO. Methyl eugenol, eugenol and linalool were the major constituents, and also detected in other *Ocimum* species, including *O. basilicum*, *O. gratissimum*, *O. campechianum*, and *O. tenuiflorum* [29,30]. Sesquiterpenes were the second most abundant class. Some of them, such as β-caryophyllene and germacrene D, are also common in other *Ocimum* species, such as *O. suave* [31]. The sesquiterpene mint sulfide was the most unusual component characterized in this EO, being identified for the first time in the genus *Ocimum*. However, previous studies on this species revealed evident compositional variation, which could be attributed to the geographical and environmental diversity [26,27,28].

This genus has multiple traditional uses in addition to its reported pharmacological properties [1,2,3]. The inhibition of cancer cell proliferation and survival are some of the main strategies of anticancer drugs [14]. In the present study, the cytotoxic effect of OFEO against one breast and two colon cancer cells was studied using the MTT method. The resulting IC50 ranged between 5.34 and 17.09 μg/mL. The colon cancer cells (HT29 and HCT116) were more sensitive to OFEO compared with the breast cancer cells (MCF7). Most importantly, OFEO showed selectivity against the three cancer cells (especially against HCT116 cells) compared with the normal cells (MRC5). Next, the HCT116 cell line was subjected to clonogenic assay, which confirmed that OFEO has a dose-dependent clonogenic effect on this cell line. Further mechanistic studies using Western Blotting and qRT-PCR showed that OFEO inhibited cell proliferation, caused cell cycle arrest, and induced apoptosis in HCT116 cells. This was evident as OFEO significantly upregulated the expression level of Caspase 3, while downregulating AKT, pAKT, PCNA, Cyclin D1, and Bcl2, all compared with GAPDH. Previously, a non-volatile metabolite, caffeic acid, isolated from *O. gratissimum* L. showed both anti-proliferative and apoptotic effects against HeLa cells [9]. However, ursolic acid, isolated from an *O. basilicum* extract, showed cell cycle effects [10]. The main constituent methyl eugenol constitutes 55.65% of the whole oil, and the activity could be attributed mainly to this compound, and methyl eugenol or the essential oils/extracts containing it were proofed for their anticancer activity; the ethanol extract *Rosa persica* roots exhibited anticancer activity against human glioblastoma (U-87-MG) and human breast cancer (MDA-MB-231) cell lines [45], and another study revealed that the combination of methyl eugenol and myricetin enhanced the anticancer activity, cell cycle arrest, and apoptosis induction of cis-platin against HeLa cervical cancer cell lines [46]. Some studies on eugenol have also revealed a potential apoptotic and anti-angiogenic effect in gastric carcinogenesis [47]. Eugenol decreased Bcl-2 and Bcl-xL expression, and increased the expression of Bax, Bid, Bad, Apaf-1, cytochrome C, and caspase-9. Eugenol induced autophagy and apoptosis in triple-negative breast cancer cells (MDA-MB-231) via pi3k/akt/foxo3a pathway inhibition [48]. Some studies have also revealed the anticancer effect of eugenol on the HCT116 cell line [49]. In the present study, the markers that upregulate the apoptosis and downregulate the proliferation were determined by Western Blot.

MIC can be used as a semi-quantitative assay, indicating the minimum concentration capable to inhibit the growth of each bacterial strain. The well diffusion method and MIC assay performed in the present study showed that OFEO had a certain antimicrobial effect on Gram-negative strains and a good inhibition activity against the Gram-positive bacteria *(S. aureus*). In this study OFEO was shown to inhibit growth of all bacteria at 250 μg/mL, except *P. aeruginosa*, which required 500 μg/mL, indicating good antimicrobial activity of this oil. The essential oil of *Ocimum* sp. has previously been reported to possess marked antimicrobial activity against a broad range of bacteria and fungi due to the presence of diverse classes of volatile constituents, such as monoterpenes (linalool, 1,8-cineole, geranial and neral), phenylpropanoids (methyl cinnamate, methyl chavicol, eugenol), and sesquiterpenes (β-selinene) [12,32,33]. Thus, OFEO has cytotoxic antiproliferative, cell cycle arrest, and apoptotic effects on HCT116 colon cancer cells. The antimicrobial effect observed on Gram-negative bacteria (*E. coli* and *K. pneumonia*) and Gram-positive bacteria (*S. aureus*) warrant further phytochemical and pharmacological investigations on the essential oil [2].

## 5. Conclusions

OFEO is rich in secondary metabolites, which possess many biological and therapeutical properties that indicate its possible use for the treatment of various ailments. It is mainly composed of small molecules, which have the ability to interact with cellular enzymes, causing upregulation of apoptotic protein and downregulation of proliferative and cell cycle proteins. Since this plant is used as a food additive, it could be recommended for use as an adjuvant cancer therapy, as well as a supplement for the prevention of malignant tumors. In addition, OFEO could be used to compose certain anti-microbial formulas to be used locally and systematically for the treatment of infectious diseases. As potential future research, OFEO could be investigated in combination with classical anticancer drugs in order to reduce their side effects and enhance their therapeutic effects.

As a limitation of our work, we can highlight some technical issues related to the formulations and the lipophilic character of the essential oil, which should imply the use of advanced formulation technologies, such as micro and nano formulations.

## Figures and Tables

**Table 1:** Sequence of primers used in qRT-PCR and their main role(s) in cancer.

Gene	Sequence	Main Role(s) in Cancer
AKT	F:GTGGCAAGATGTGTATGAG R:CTGGCTGAGTAGGAGAAC	Cell proliferation and survival
pAKT	F: GGACAAGGACGGGCACATTA R: CGACCGCACATCATCTCGTA	
PCNA	F: AAGGAGGATGAAGCGGTAACAAT R: GTCTTGGACAGAGGAGTGGC	Cell cycle arrest
cyclinD1	F: CCAGCCGCAATGCTGTAG R: TTGGGACGCCTCAGCTAAG	
Bcl2	F: CTCTCGTCGCTACCGTCGCG R: AGGCATCCCAGCCTCCGTTATCC	Anti-apoptosis
Caspase 3	F: ACATGGAAGCGAATCAATGGACTC R: AAGGACTCAAATTCTGTTGCCACC	Apoptosis
GAPDH	F: AGGTCGGTGTGAACGGATTTG R: TGTAGACCATGTAGTTGAGGTCA	House keeping

**Table 2:** Complete chemical composition of the EO of *O. forskolei* obtained from the flowering aerial parts.

Compounds	l.r.i ^1^	Class	Relative Abundance (%) ± SD (*n* = 3)
myrcene	991	mh	0.1 ± 0.01
*(Z)*-β-ocimene	1036	mh	0.1 ± 0.01
*(E)*-β-ocimene	1047	mh	2.5 ± 0.18
linalool	1101	om	9.8 ± 0.09
α-terpineol	1191	om	0.2 ± 0.01
eugenol	1357	pp	12.1 ± 0.45
α-copaene	1376	sh	0.2 ± 0.02
β-bourbonene	1385	sh	0.1 ± 0.01
β-elemene	1392	sh	0.2 ± 0.04
methyl eugenol	1405	pp	56.6 ± 0.94
β-caryophyllene	1419	sh	2.4 ± 0.17
*trans*-α-bergamotene	1436	sh	1.1 ± 0.10
α-guaiene	1439	sh	0.9 ± 0.09
α-humulene	1453	sh	1.3 ± 0.09
*(E)-*β-farnesene	1458	sh	0.7 ± 0.04
germacrene D	1481	sh	3.5 ± 0.26
β-selinene	1486	sh	0.5 ± 0.04
α-selinene	1495	sh	0.3 ± 0.02
bicyclogermacrene	1496	sh	0.2 ± 0.03
aciphyllene	1499	sh	0.2 ± 0.03
α-bulnesene	1505	sh	2.2 ± 0.14
*trans*-γ-cadinene	1514	sh	0.1 ± 0.02
δ-cadinene	1524	sh	0.4 ± 0.03
*cis*-sesquisabinene hydrate	1544	os	0.1 ± 0.01
germacrene D-4-ol	1574	os	0.2 ± 0.02
caryophyllene oxide	1582	os	0.3 ± 0.02
humulene oxide II	1608	os	0.1 ± 0.01
T-cadinol	1641	os	0.9 ± 0.01
β-eudesmol	1649	os	0.4 ± 0.02
α-cadinol	1654	os	0.7 ± 0.02
β-sinensal	1695	os	0.9 ± 0.16
mint sulfide	1735	sh S	0.4 ± 0.03
phytol	2112	od	0.5 ± 0.08
Monoterpene hydrocarbons (mh)			2.7 ± 0.20
Oxygenated monoterpenes (om)			10.0 ± 0.09
Sesquiterpenes hydrocarbons (sh)			14.2 ± 0.99
Oxygenated sesquiterpenes (os)			3.6 ± 0.18
Sulfur sesquiterpene hydrocarbons (sh S)			0.4 ± 0.03
Oxygenated diterpenes (od)			0.5 ± 0.08
Phenylpropanoids (pp)			68.7 ± 1.39
Total identified (%)			100.0 ± 0.01

^1^ Linear retention index on a HP 5-MS capillary column.

**Table 3:** Cytotoxic activity of OFEO and doxorubicin against three cancer cell lines and normal one.

Component	MCF7	HT29	HCT116	MRC5
OFEO	17.09 ± 1.96	6.66 ± 0.17	5.34 ± 1.64	24.80 ± 1.12
Doxorubicin	2.09 ± 0.17	1.50 ± 0.21	3.33 ± 0.75	2.50 ± 0.14

**Table 4:** Selectivity of OFEO and doxorubicin against the cancer cells compared with normal MRC5 fibroblasts.

Component	Selectivity Index *^a^*		
	MCF7	HT29	HCT116
OFEO	1.45	3.72	4.64
Doxorubicin	1.19	1.66	0.75

*^a^* Selectively index (SI) = IC_50_ value against the normal MRC5 cells divided by the IC_50_ value against the corresponding cancer cell line. The experiment was repeated three times.

**Table 5:** Means of inhibition zone in mm for each bacterial strain with the three solutions and positive control (discs of Amikacin for G −ve bacteria and Amoxicillin for G +ve bacteria).

Treatment	*E. coli*	*K. pneumonia*	*P. aeruginosa*	*S. aureus*
OFEO	15 ± 0.50	15 ± 1.00	15 ± 0.50	13 ± 0.50
Amikacin	18 ± 1.01	20 ± 1.01	22 ± 1.01	-
Amoxicillin	-	-	-	15 ± 2.01

**Table 6:** The mean of the MIC assay for the OFEO solution with the bacterial reference strains used in this study. Each concentration is shown in μg/mL.

Bacteria Strains	*E. coli*	*K. pneumonia*	*P. aeruginosa*	*S. aureus*
OFEO Concentration (μg/ml)	250	250	500	250

## Data Availability

All relevant data are contained in the present manuscript.

## Abbreviations

The following abbreviations were used: OFEO, *Ocimum forskolei* essential oil; MCF-7 Michigan Cancer Foundation-7 breast cancer; HT-29, human colon cancer cell line; HCT116, human colorectal carcinoma cell line; MRC-5 (Medical Research Council cell strain 5) fibroblasts; MTT assay, 3-(4,5-dimethylthiazol-2-yl)-2,5-diphenyltetrazolium bromide; qRT-PCR, Real-Time Quantitative Reverse Transcription PCR.
